# Tregs combined with mature donor T cells hasten immune reconstitution without triggering GvHD in HLA haploidentical transplantation

**DOI:** 10.1186/ar3410

**Published:** 2011-09-16

**Authors:** Mauro Di Ianni, Franca Falzetti, Massimo F Martelli

**Affiliations:** 1 Hematology and Clinical Immunology Section, Department of Clinical and Experimental Medicine, University of Perugia, Italy; 2 Weizmann Institute of Science, Immunology Department, Rehovot, Israel

## 

Haploidentical transplantation, with extensive T cell depletion to prevent GvHD, is associated with a high incidence of infection-related deaths. The key challenge is to improve immune recovery with allogeneic donor T cells without triggering GvHD. As T regulatory cells (Tregs) controlled GvHD in preclinical studies, the present phase I/II clinical trial evaluated the impact of early infusion of donor CD4/CD25+ Tregs, followed by an inoculum of donor mature T cells (Tcons) and positively immunoselected CD34+ cells. Twenty-eight patients (median age 41, range 21-60) were enrolled from September 2008 onwards; 22 had AML (10 in CR1 at high risk, 10 in ≥CR2 and 2 in relapse), 5 had ALL (4 in CR1; 1 in relapse) and 1 had high grade NHL in relapse. Conditioning was: 8 Gy single fraction TBI, thiotepa (4 mg/kg × 2), fludarabine (40 mg/m^2^ × 5), cyclophosphamide (35 mg/kg × 2). All patients received immunoselected Tregs (CliniMACS, Miltenyi Biotec) (23/28 2 × 10^6^/kg bw; 5/28 4 × 10^6^/kg bw) and 4 days later positively immunoselected CD34+ cells (median 8.2 × 10^6^/kg bw, range 5.0-19.1) together with Tcons (4/28 0.5 × 10^6^/kg bw; 17/28 1 × 10^6^/kg bw; 5/28 2 × 10^6^/kg bw; 2/28 did not receive Tcons). CD4/CD25^+ ^Tregs (purity 92.7 ± 2.1) consisted of 33.6% ± 13.1 CD25^high^; 58.1% ± 6.6 CD25^int^; 5.8% ± 2.5 CD25^low^; 65.7% ± 11.8 FoxP3; 17.4% ± 7.2 CD127 (mean ± SD). No GvHD prophylaxis was administered. 26/28 patients engrafted. No GvHD developed in 24/26 patients, 2 developed ≥ grade II GvHD. Ten patients died (3 VOD, 2 fungal pneumonia, 1 bacterial sepsis, 1 CNS aspergillosis, 1 systemic toxoplasmosis, 1 adenoviral infection, 1 MOF). CD4 and CD8 counts reached, respectively, 50/μL medianly on days 34 (range 19-63 days) and 24 (range 15-87); 100/μL medianly on days 47 (range 28-100 days) and 34 (range 19-95); 200/μL on days 70 (range 41-146 days) and 61 (range 21-95). A wide T-cell repertoire developed rapidly with high frequencies of specific CD4+ and CD8+ for opportunistic pathogens. Episodes of CMV reactivation were significantly fewer than after our "standard haplo" transplants. In KIR ligand-mismatched transplants, speed of NK cell reconstitution/maturation and size of donor vs recipient alloreactive NK cell repertoires were preserved. In conclusion, in the setting of haploidentical transplantation infusion of Tregs makes administration of a high dose of T cells feasible for the first time. This strategy provides a long-term protection from GvHD and robust immune reconstitution.

